# Supplementary material for: Design of a clinical balance tool for fall risk assessments: A development and usability study
Source: PLoS One. 2025 Feb 21;20(2):e0302080. doi: 10.1371/journal.pone.0302080 (PMC11844839; doi:10.1371/journal.pone.0302080)
Supplement: S1 Table — (DOCX) [file pone.0302080.s001.docx]

**S1 Table**. SUS quartile ranges and scores.

| **SUS Score** | **Letter Grade** | **Adjective Rating** |
| --- | --- | --- |
| Above 80.3 | A | Excellent |
| Between 68 and 80.3 | B | Good |
| 68 | C | OK |
| Between 51 and 67 | D | Poor |
| Below 51 | F | Awful |
