# Supplementary material for: Design of a clinical balance tool for fall risk assessments: A development and usability study
Source: PLoS One. 2025 Feb 21;20(2):e0302080. doi: 10.1371/journal.pone.0302080 (PMC11844839; doi:10.1371/journal.pone.0302080)
Supplement: S2 Table — (DOCX) [file pone.0302080.s002.docx]

**S2 Table**. Barriers to the use of guidelines based on the Cabana Framework.

| Factor in Cabana Framework | Knowledge | Attitudes | Behavior |
| --- | --- | --- | --- |
|  | - Lack of awareness of guidelines - Lack of familiarity with guidelines | - Lack of agreement with guidelines - Lack of outcome expectancy - Lack of self-efficacy - Lack of motivation | - Patient factors   -Inability to reconcile patient preference with guidelines  -Recommendations   - Guideline Factors   -Guidelines  -Characteristics  -Presence of contradictory guidelines   - Environmental Factors   -Lack of Time  -Lack of Resources  -Organizational constraints  -Lack of reimbursement  -Perceived increase in malpractice liability |
| Results | - Some physicians were aware of the applicable guidelines - Physicians and nursing staff exhibited high measurement for lack of familiarity with the guidelines | - Positive attitudes toward guidelines - Preference for recommended treatment or elements of the guidelines | - The patient doesn’t have a chronic illness (sports medicine) - The patient had a hard time walking or was obese (family medicine) - Practice/clinic (inpatient or outpatient setting) - Academic/Private - Sports medicine (doesn’t take all vital signs) - How guidelines are implemented in clinical settings - Lack of time (insufficient time to inform or negotiate with patients) - Guidelines (unclear or impractical to use) |
