# Supplementary material for: Design of a clinical balance tool for fall risk assessments: A development and usability study
Source: PLoS One. 2025 Feb 21;20(2):e0302080. doi: 10.1371/journal.pone.0302080 (PMC11844839; doi:10.1371/journal.pone.0302080)
Supplement: S3 Table — (DOCX) [file pone.0302080.s003.docx]

**S3 Table**. Participant descriptions of barriers to use of guidelines based on the Cabana Framework.

| Title | Snippets |
| --- | --- |
| Knowledge | *“I’m aware of them. I’m probably not as well versed as I should be.”* (Lack of familiarity)  *"I know they're out there; I couldn't tell you exactly what they are though."*  *“I do know that they have them. I’m not familiar with the specifics of them but I know that they’re available. I know where to find them.”* (Lack of awareness) |
| Attitudes | *“I do have concerns about it. Because, I don’t have it in front of me, its medicine and its age, and I think some people score higher than I would expect them to have a problem and I sometimes don’t think, because of the medicines, the medicines are very high I think on the list. Which does make sense, but I... Yeah, yeah in some ways I don’t [inaudible] that, yeah. It’s not perfect, at all.”* (Lack of agreement) |
| Behaviors | *“Some patients get no vitals, if they're coming just in for an injection and they're just a follow up patient, I don't get vitals 'cause it doesn't really help the visit.”* (Sports Medicine-doesn’t take all vital signs)  *"I don't have a formal assessment. It's pretty much by history and what I see on exam...Well, history, if they tell me that they're falling, or they say that they're unsteady on their feet, or if they have significant joint pain, I ask them a little bit more about that if they fall. On exam, I see mostly lower extremities so I have all of them stand up and try to balance on one leg, or do a one leg squat, and you can tell pretty quickly if they're unsteady, and then watch them walk."* (Guidelines-unclear or impractical to use)  *“We actually have a falls risk calculator, that we use for our Medicare patients, or basically in our office we do it for anybody who’s over 65, so there is a falls risk calculator that my medical assistant asks questions on...We do that, 65 and above, on their physical exams.”* (How guidelines are implemented in clinical settings) |
| Patient Factors | *Interviewer: "Yeah. So, remind us on what sorts of patients might you do vitals or skip certain parts."*  *Interviewee: "Mm-hmm (affirmative). The only ones, at least for our surgeons that we need to do vitals on, are the people that are going in for surgery.”* (sports medicine-patient doesn’t have a chronic illness)  *“The type of patients that I see, most of them don't have any fall issues. I assess them by looking at the patient, well examining them. I will see how they walk from chair to examining table. And that's how I would observe.”*  *"I keep a close eye on patients when they're walking, so that way I can assess how well they walk. And how they maneuver throughout the room once they get to the room. Also, we have a falls risk assessment, where we ask questions…"* |
