# Supplementary material for: Design of a clinical balance tool for fall risk assessments: A development and usability study
Source: PLoS One. 2025 Feb 21;20(2):e0302080. doi: 10.1371/journal.pone.0302080 (PMC11844839; doi:10.1371/journal.pone.0302080)
Supplement: S4 Table — (DOCX) [file pone.0302080.s004.docx]

**S4 Table**. Recommended tool changes and modifications from the first round of interviews.

| Topic | Recommendation (snippets) | Modifications Made |
| --- | --- | --- |
| Features favored by participants with additional requests | *"Maybe it would be better to have, instead of an iPad, another connected screen, like a small screen with a push button. And as far as storing it, I don't think it would necessarily have to be stored on the device, and I think it'd probably be complicated to have it sent from the device to the ..."*  *“No, I think, you know, as far as just like, nope that’s fine. Yeah. I mean, are you, you’re not putting the display down there, right? It’s gonna be up on a...? You’re not sure yet... Up high is always good, the medical assistants can get older, nobody wants to lean over.”*  *"You could add outlines of feet on there, because some of these people might say, "Okay, you have to be on both sides of the green but just touching. That might be somewhat hard, so maybe if you had a foot outline, like in airport scanners"*  *"Just with the critical one. I know the number but maybe just like it changes to red or something to alert people that it's critical. Just like how you do it now, green is go so green is good, yellow is maybe color-coded yellow is caution and red is failing."*  *"Oh, I, an audible beep at the end might be good, because then I could watch the individual, I kept, you know towards the end I was watching the clock, splitting time between watching the clock and watching the patient because I wouldn’t want them to fall, but at the same time I didn’t know if it was going to stop, and we’d just be sitting there for...No, just the end, just so that I can watch the person the whole time, I can hit the start button and then not pay attention to that until the end.”* | - Options for the tablet to be at eye level - Two options for foot outlines or guidelines - Color coordination with a low, moderate, and high risk of falls on the instruction sheet - The scale is smaller and lower to the ground - Audible beep at the end of the 30-second test - Markings to guide placement of feet |
| Missing information | Sync information directly into IHIS [the electronic health record] | - No modifications made; best to write the number down and put it directly into vital signs |
| Recommended changes to information or presentation | *“Yeah, so I would say, if it could read in a shorter amount of time, with the scale if it was very consistent, and I think it read to the tenth of a point, but we need you know at least to the tenth.”*  *"...well, the patient has their eyes closed. I was thinking if there was something that the patient could actually see the countdown so they would know what was left, but their eyes are closed, so scratch that. Yeah, just something with the time."*  *“Probably time or, the time on the scale or if, if the time isn’t something that could be adjusted, maybe you know, being able to add ‘30 seconds left’, ‘15 seconds left’, just being able to at least update the patient on how long, especially if their eyes are closed. Just so they have an awareness of the time.”*  *"...I mean, even if I'm holding my computer, I'm going to see that countdown going. Where I can say, "you are about halfway done." You are doing a great job." Or if they were to start to move, you know, their hands or get fidgety or something like that, you know. Just to be able to say something to them where you wouldn't have to start the test over..."*  *"Taking shoes on and off could be a little bit of a challenge I guess." "Well, this seems like a long time to stand there waiting for that to happen. If it only took like 20 seconds, it seems like that would feel less. We'd probably do it more frequently than if it took a full minute I guess."* | - Changed the weight readout to show a tenth of a point - With the patient’s eyes closed, support staff can talk with patients, telling them how much time is left - Support staff can hold out an arm to catch the patients if they begin to swerve - Display can hold the weight and balance score until support staff clears the data |
