# Supplementary material for: Design of a clinical balance tool for fall risk assessments: A development and usability study
Source: PLoS One. 2025 Feb 21;20(2):e0302080. doi: 10.1371/journal.pone.0302080 (PMC11844839; doi:10.1371/journal.pone.0302080)
Supplement: S5 Table — (DOCX) [file pone.0302080.s005.docx]

**S5 Table**. Emerging themes with supporting snippets.

| Providers assess fall risk individually and as a team. The team process involves multiple inputs throughout the patient visit, with the primary care provider at the center of the process (action step). | *“The PCA normally relays that to us, saying, ‘this person was very unstable, they almost fell in the hallway, or I’m worried about them falling.’ I don’t believe there’s documentation, so it’s verbal.”*  *“It’s mainly just each individual part of the team doing a mini-assessment during the visit, doing an assessment and then recording that as such in the chart.”*  *“The nurse notes it will be in the review of systems, which I will then elaborate on in my HPI if I think it’s a problem or complaint.”* |
| --- | --- |
| Different types of assessments were done. Assessment techniques include observation, patient reports, provider questioning, and use of evidenced-based algorithms or segments of algorithms, specific testing techniques, and tools. | *“We actually have a falls risk calculator”*  *“Let's say that the patient came in for syncope, which I had a recent patient come in for that. So, then I asked, "Have you fallen in the last few days when you collapsed, was there a fall involved?" And then we would document that in the chief complaint.”*  *“Yeah, observe I guess, yes. I observe them.*  *“I mean sometimes I use a reflex hammer. I’ll use a monofilament especially if I’m worried about the feeling in the feet definitely. But there’s a lot of good just run of the mill, good for the whole exam skills that don’t need a lot of necessary like complex equipment to really assess a lot of things that I feel is necessary.”* |
| Documentation of fall risk assessments may be fragmented due to the use of verbal communication, and notes outside of the EHR. Some of the assessment is not in the medical record. | *“They say out loud these things. So, they’ll say, ‘her blood pressure was really bad’ or ‘she almost fell’ or ‘I’m worried about her, she doesn’t look good.’ And so, they’ll say those things as they drop the chart.”*  *“But, the little things about, so, the RN who writes the no falls risk, if she’s saying they went to the ED in December since we saw them last, etc. She doesn’t put that anywhere.”*  *“So, I guess maybe talking with Dr. - you know, straight with [physician's name], would be outside of the record would be another thing that we do. But she and I are always in really good communication.”* |
| Benefits of fall risk assessments were articulated by multiple team members. | *“Figuring out what we need to do for the fall, or why they're having the falls and then trying to get them into the right treatments.*  *“If by, by putting me into a certain pathway then I can be much more focused during my exam and I can, and hopefully be able to come up with a better differential or actually diagnosis so that we can get a patient to the appropriate treatment to improve their fall or balance issue.”* |
| Two negative aspects of the algorithms were described as the use of age and medications. This results in age and medication driving the risk more, which may not be clinically appropriate. | *“I do, I guess I do have concerns about it. Because, I don’t have it in front of me, its medicine and its age.”*  *“I think some people score higher than I would expect them to have a problem and I sometimes don’t think, because of the medicines, the medicines are very high I think on the list. Which does make sense, but I... Yeah, yeah in some ways I don’t [inaudible] that, yeah. It’s not perfect, at all.”*  *“So, I would think that it would be more than just a specific age population.”* |
| Barriers to translating fall risk evidence into clinical practice include a lack of awareness of the guidelines and a lack of use of the current guidelines. | *“I do know that they have them. I’m not familiar with the specifics of them but I know that they’re available. I know where to find them.”*  *But I’m not familiar with any of them in-depth, at all.”*  *“I’ve heard of them, but I honestly am not overly familiar with the content or would be able to reiterate it.”*  *“I think the trouble is fitting them into your clinic, fitting them into your schedule, making them work in a workflow.”*  *“I’m aware of them. I’m probably not as well versed as I should be.”* |
