# Supplementary material for: Design of a clinical balance tool for fall risk assessments: A development and usability study
Source: PLoS One. 2025 Feb 21;20(2):e0302080. doi: 10.1371/journal.pone.0302080 (PMC11844839; doi:10.1371/journal.pone.0302080)
Supplement: S6 Table — (DOCX) [file pone.0302080.s006.docx]

**S6 Table**. Work processes associated with obtaining vital signs.

| Vital Signs Taken | 100% (18/18) of the clinics measured weight and blood pressure |
| --- | --- |
|  | 94% (17/18) of the clinics measured height and pulse |
|  | 33% (6/18) of physician extenders and physicians did not take vital signs for every patient at each visit to the sports medicine clinic, visit for a sports-related injury, or follow-up appointment |
|  | All (4) non-sports medicine clinics checked these vital signs, whereas (1) sports medicine clinic only checked vitals that were needed (weight, height, pulse) |
| Location | 83% (15/18) of vital signs were taken at a vital station in the hallway |
|  | 67% (12/18) were taken in the exam room |
|  | 5% (1/18) were taken in a triage room |
| Weight | 100% (18/18) of participants measured weight |
|  | 22% (4/18) of participants asked patients to take their shoes off when weight was taken |
