# Supplementary material for: Design of a clinical balance tool for fall risk assessments: A development and usability study
Source: PLoS One. 2025 Feb 21;20(2):e0302080. doi: 10.1371/journal.pone.0302080 (PMC11844839; doi:10.1371/journal.pone.0302080)
Supplement: S7 Table — (DOCX) [file pone.0302080.s007.docx]

**S7 Table**. Example snippets from the first round of interviews.

| Evaluation Area | Positive Snippets Related to Tool Design | Requirements, Recommendations, and Areas for Tool Improvement |
| --- | --- | --- |
| Clinical content (assessment) | *“The smaller the size, the better it will be. That sounds perfect to me, and we can do weight and balance at the same time.”*  *"Well, like I said, it's not a tall as the scale, so it's easier to step on and you get more information out of it as opposed to just a traditional scale…It's very easy to use. It's pretty easy just to understand just the directions you had to give to the patient. It looks like - I mean, it doesn't take up that much space."* | *“Yeah, so I would say, if it could read in a shorter amount of time, with the scale if it was very consistent, and I think it read to the tenth of a point, but we need you know at least to the tenth.”*  *"Oh, I, an audible beep at the end might be good, because then I could watch the individual…”*  *"You could add outlines of feet on there, because some of these people might say, "Okay, you have to be on both sides of the green but just touching. That might be somewhat hard, so maybe if you had a foot outline, like in airport scanners."*  *"Yeah. No, I think it'd be fine. They wouldn't be able to get their height assessed while they're eyes or closed and they're doing the balance, but, yeah, I think it would make the most sense just to have ... Patients are already comfortable with standing on a scale and getting their height determined. In most facilities, that's how it goes, so I think the same kind of thing, saying, "Oh, this is our new scale, and it also does balance."* |
| Usefulness of the decision support tool (cross-issue) | *“Oh, well you can always use it because it’s a scale, right? Right? I mean, there's no reason not to use it. Except for the cost. But if it’s a scale there’s no reason not to use it.”*  *"It's very similar to the scale I use now. So, there's not really much different as that goes. There's not much of a platform up so if the patient was unsteady. It's not like a mountain fall down. Just very similar to what we use in a regular weight to scale now."* | *"...I might not say all my patients but you know my hardest patients so like at least all of my Medicare patients and maybe like you know...Maybe I would just use it on 65 and older and anyone who might be just complex so that, plus I’m at a higher risk for those types of issues. I might, you probably might state something like that so.”*  *“I mean, I think I could see it being used on every patient versus like if we could identify a higher risk population…”* |
| Human-computer interface (informatics and managing) | *“I like the interpretation result, I think that’s a good thing to know, especially with our patient population, just what it does with that to calculate the score… but even that blue area seems pretty good for a size, big enough size to stand for patients… And I like how easy it is to use, just step right on and just wait for the minute.”* | "*I don't know how it would be implemented, but if it integrated in IHIS, that could even be the first question, shoes on or off. And you don't even ask them, but something you mark in the chart, so it would be there." "Well, not if it's something that can automatically populate and go in there. I mean, if they have to click too many buttons and get to too many places, yeah, that would slow it down even more."* |
