# Supplementary material for: Design of a clinical balance tool for fall risk assessments: A development and usability study
Source: PLoS One. 2025 Feb 21;20(2):e0302080. doi: 10.1371/journal.pone.0302080 (PMC11844839; doi:10.1371/journal.pone.0302080)
Supplement: S8 Table — (DOCX) [file pone.0302080.s008.docx]

**S8 Table**. Example snippets from the second round of interviews.

| Evaluation Area | Positive Snippets Related to Tool Design | Requirements, Recommendations, and Areas for Tool Improvement |
| --- | --- | --- |
| Clinical content (assessment) | *"I especially like that I can tell how much time is left, I believe last time I was not allowed to say anything, and it seemed like an eternity even though it was only 30 seconds. I liked that and smoothness-wise, we already have patients take off their shoes and get on the scale so to add that into the flow I don't think would be that much more difficult."*  *"Well, top three things is it gives weight at the same time and it's convenient to use. Instructions are simple. I think you can train medical assistants to use it. It's not too complicated."*  *"I think the fact, well... It's actually smaller than when, when he had sort of described... Essentially a lot smaller than I expected it to be, even though sort a small flat. So, it's sort of nice, and I think in terms of user interface that seems fairly easy. Now, does it always happen... Is it planned to always be connected via sort of data connection to like a tablet device or would it be sort of integrated in like..."* | *“…I'm trying to think of any improvement. Our scale that we use right now also has a handle next to it so people can hold on. So maybe if there was some kind of bar around it that someone could grab if they got dizzy. I guess that could be an improvement. What else? I guess that's it. I can't really think of too much else.”*  *"Okay. I know it's counter-intuitive, but having something in case you do need a patient to grab onto something where they're not able to complete the task, it'd be good to have around there they're able to grab onto, that you're not... Your only source of them falling onto the ground. If there's any way to make this thing be rapid, like a two second test, that would be even better. I know that's impossible, but... And then met. Maybe a little bit of a wider base just for some of the population that we have here, but if they're able to get on that, no problem. I don't think that's really too much of an issue."* |
| Usefulness of the decision support tool (cross-issue) | *"I think in terms of workflow it would be pretty easy minus the added step. But I mean, like I said, we've got a patient on a scale every time they come and get their vitals. So, in terms of the workflow, like no matter which clinic you're in, they're probably going to go into a vitals room or hallway and get their weights anyways. So just adding that to that, I think it's definitely reasonable."*  *"It's helpful, it gives some more objective data that the patient and me could both visually look at and see that progress was made. And it's quick, I think that's helpful. Easy to use."*  *"No, I think it'd be easy. There's already a weight machine, so it's much different than them having to stand on another machine to get the weight and then it's small. It fits well. It's short duration overall. Yeah."* |  |
